# Demethylbelamcandaquinone B (Dmcq B) Is the Active Compound of *Marantodes pumilum* var. *alata* (Blume) Kuntze with Osteoanabolic Activities

**DOI:** 10.3390/molecules23071686

**Published:** 2018-07-11

**Authors:** Haryati Ahmad Hairi, Jamia Azdina Jamal, Nor Ashila Aladdin, Khairana Husain, Noor Suhaili Mohd Sofi, Norazlina Mohamed, Isa Naina Mohamed, Ahmad Nazrun Shuid

**Affiliations:** 1 Department of Pharmacology, Faculty of Medicine, Preclinical Building, Universiti Kebangsaan Malaysia, Jalan Yaacob Latiff, Bandar Tun Razak, Cheras, 56000 Kuala Lumpur, Malaysia; haryatiahmadhairi@gmail.com (H.A.H.); noorsuhaili.sofi@yahoo.com (N.S.M.S.); azlina@ppukm.ukm.edu.my (N.M.); isanaina@yahoo.co.uk (I.N.M.); 2 Faculty of Pharmacy, Universiti Kebangsaan Malaysia, Jalan Raja Muda Abdul Aziz, 50300 Kuala Lumpur, Malaysia; jamia@ukm.edu.my (J.A.J.); ashi_qu88@yahoo.com (N.A.A.); khairana@ukm.edu.my (K.H.)

**Keywords:** *Marantodes pumilum* var. *alata*, osteoblast, anabolic effects, phytoestrogen, estrogen receptor

## Abstract

Phytoestrogens have attracted considerable attention for their potential in the prevention of postmenopausal osteoporosis. Recently, a phytoestrogen-rich herbal plant, *Marantodes pumilum* var. *alata* (Blume) Kuntze was reported to protect against bone loss in ovariectomized rat. However, the bioactive compound responsible for these effects and the underlying mechanism were not known. Through bioassay-guided isolation, demethylbelamcandaquinone B (Dmcq B) was isolated and identified from *Marantodes pumilum* var. *alata* leaf extract. In terms of its bone anabolic effects, Dmcq B was at par with 17β-estradiol (E2), in promoting the proliferation, differentiation and mineralization of osteoblast cells. Dmcq-B increased early differentiation markers, collagen content and enzymatic ALP activity. It was demonstrated to regulate BMP2 signaling pathway which further activated the transcription factor, osterix. Subsequently, Dmcq B was able to increase the osteocalcin expression which promoted matrix mineralization as evidenced by the increase in calcium deposition. Dmcq B also reduced the protein level of receptor activator of NF-κβ ligand (RANKL) and promoted osteoprotegerin (OPG) protein expression by osteoblast cells, therefore hastening bone formation rate by decreasing RANKL/OPG ratio. Moreover, Dmcq B was able to increase ER expression, postulating its phytoestrogen property. As the conclusion, Dmcq B is the active compound isolated from *Marantodes pumilum* var. *alata* leaves, regulating osteoanabolic activities potentially through the BMP2 and ER signaling pathways.

## 1. Introduction

Bone remodeling is a dynamic process that maintains the balance between the bone formation and resorption processes. The sequence starts when osteoclasts resorb old mineralized bone, followed by new bone formation and mineralisation by osteoblasts. This allows the bone to adapt to biomechanical forces, repair bone matrix microtraumas and hence improve bone structure from time to time [1]. Estrogen is the foremost modulator of bone remodeling. The decline in estrogen during menopause is the starting point for the disturbance of the bone remodeling cycle, which finally leads to acute bone loss [2]. Osteoporosis has become a public health threat, predominantly in post-menopausal women. Estrogen replacement therapy (ERT), the linchpin of osteoporosis treatment is inimitably able to increase bone formation rate. However, long term exposure to synthetic estrogen may cause uterine and mammary gland hyperplasia, leading to increased risk of breast cancer, uterine cancer, and increased risk of cardiovascular disease [3].Selective estrogen receptor modulators (SERM) drugs such as tamoxifen [4], raloxifene [5], bazedoxifene [6] and lasofoxifene [7] were developed to produce desirable estrogenic effects on specific organs with less side effects on other organs, however, it was found that these drugs may produce similar side effects to ERT. 

Phytoestrogens are non-steroidal compounds derived from plants and have activities that can mimic endogenous estrogen actions. Based on their chemical structure which typically has a ring and a pair of phenolic hydroxyl groups, phytoestrogens can bind to estrogen receptors (ER), either at ERα or ERβ [8]. Recently, the interests in assessing phytoestrogens as anti-osteoporotic agents have surged due to their presumed safety as natural products. Isoflavones, primarily genistein and daidzein, are exemplary phytoestrogens which have potential beneficial effects on bone and were shown in several clinical trials in menopausal women to have fewer to no side effects [9,10]. Therefore, they are targets of great interest for potential use in osteoporosis prevention and/or therapy. Phytoestrogens can be classified into several groups, including flavonoids (kaempferol and quercetin) [11,12], isoflavones, lignans (enterolactone and enterodiol) [13], coumestan (coumestrol) [14] and stilbene (resveratrol) [15]. They all exert beneficial effects on bone through estrogen receptor pathway.

*Marantodes pumilum* (Blume) Kuntze (synonym: *Labisia pumila* (Blume) Fern-Vill) is a natural herbal plant which belongs to the family Primulaceae. It is locally known as Kacip Fatimah and is one of the most frequently used medicinal plants in Malaysia. Traditionally, decoctions of *M. pumilum* have been used by the Malay women as an energy drink, to relieve pain during menstruation, to induce and facilitate labour, and to regain body strength during confinement [16]. *M. pumilum* was found to contain mainly triterpenoid saponins and alkylated phenolics [17], methyl gallate [18], flavonoids and phenolic compounds [19], dialkylated benzoquinones, dialkylated dibenzofuran and dexyloprimulanin [20], alkyl resorcinols and dimeric benzoquinone derivatives [21]. Research on *M. pumilum* has expanded and it was proven to possess antioxidant, antimicrobial [22], anti-inflammatory [23] and antinociceptive [24] activities. In addition, Husniza et al. [25], showed that a water extract of *M. pumilum* inhibited 17β-estradiol (E2) binding to antibodies raised against it, which resembles the effects of natural hormones such as estrone and estriol. *M. pumilum* was also able to increase estradiol level and suppress follicle stimulating hormone (FSH) and luteinizing hormone (LH) levels, therefore exhibiting similar effects to estrogen therapy [26]. It was also proven to counteract various estrogen-deficiency related diseases such as insulin resistance [27], cardiovascular diseases [28] and osteoporosis [29]. In view of the strong evidences that *M. pumilum* possesses phytoestrogenic effects, it is postulated that this plant has a favourable effect in postmenopausal women in terms of protection against osteoporosis. 

A growing number of in vivo studies demonstrated that *M. pumilum* exerts significant bone protective effects in estrogen-deficient rat model. A preliminary study from Shuid et al. [30] showed that aqueous extract of *M. pumilum* at the dose of 17.5 mg/kg given to ovariectomized rats for eight weeks exhibited higher osteocalcin, a marker of bone formation, and reduced collagen type 1 cross-linked C-telopeptide (CTX), a marker of bone resorption. Based on histomorphometric analysis, *M. pumilum* was found to increase the number of osteoblasts on bone surfaces and promote bone formation [31]. In terms of gene expression, Fathilah et al. [32] found that the aqueous extracts of *M. pumilum* were able to increase OPG and decrease RANKL expressions, and therefore can inhibit the activation of osteoclastogenesis. Micro-CT 3D analysis revealed that *M. pumilum* extracts were able to increase bone volume and trabecular bone microarchitecture in ovariectomized rats [29]. In addition, Effendy et al. reported that *M. pumilum* extract increased the levels of antioxidant enzymes, superoxide dismutase (SOD) and glutathione peroxidase (GPx). At the same time, *M. pumilum* extract reduced the level of malondialdehyde (MDA), the main product of lipid peroxidation [33]. These studies have suggested that *M. pumilum* might contain phytoestrogen compounds, a group of plant-derived substances that are structurally or functionally similar to estrogen. However, the phytoestrogen compound(s) of *M. pumilum* responsible for these effects have not been identified and the mechanisms involved have not yet been investigated. 

In our continuing efforts to explore the pharmacological values of *M. pumilum*, for the first time the present study isolated the phytoestrogenic compound in *M. pumilum* var. *alata* that has stimulatory effects on osteoblast proliferation and differentiation. This active compound was isolated using a bioassay-guided procedure and viability cells assay. The early phenotypic markers of osteoblast differentiation, collagen synthesis and enzymatic activity of alkaline phosphatase (ALP) were also measured. To evaluate the mechanisms and time frame for differentiation of osteoblast cells, transcription of osteoblastogenesis-related genes such as bone morphogenetic protein (BMP2), osterix (Osx) and osteocalcin (Ocn) were measured and confirmed with protein expression. In addition, RANKL/OPG ratio was assessed in this study to determine the extent to which the active compound(s) of *M. pumilum* var. *alata* can inhibit osteoclastogenesis. Lastly, to determine whether the active compound of *M. pumilum* var. *alata* have direct effects on ER, binding to the ERα and/or ERβ on osteoblast cells was studied. The present findings will shed light on the understanding of the active compound of *M. pumilum* var. *alata* and their effect on osteoblast differentiation and mineralization. This may provide a potential new therapeutic option for the prevention or treatment of osteoporosis.

## 2. Results

### 2.1. Active Compound of M. pumilum var. alata

**Figure 1:**
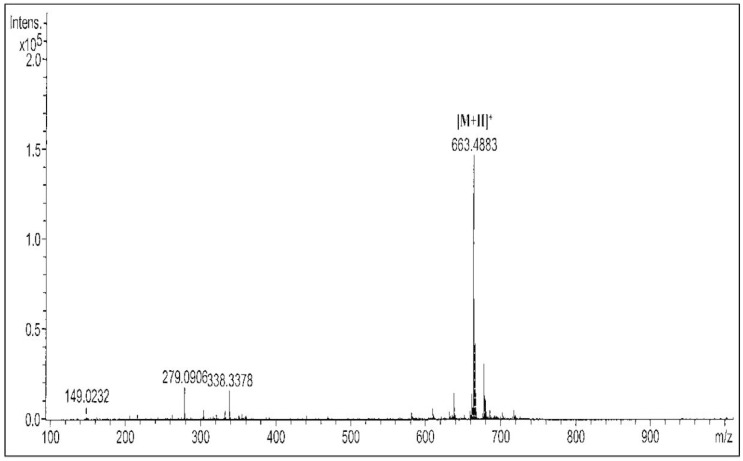
Mass spectrum of demethylbelamcandaquinone B.

**Figure 2:**
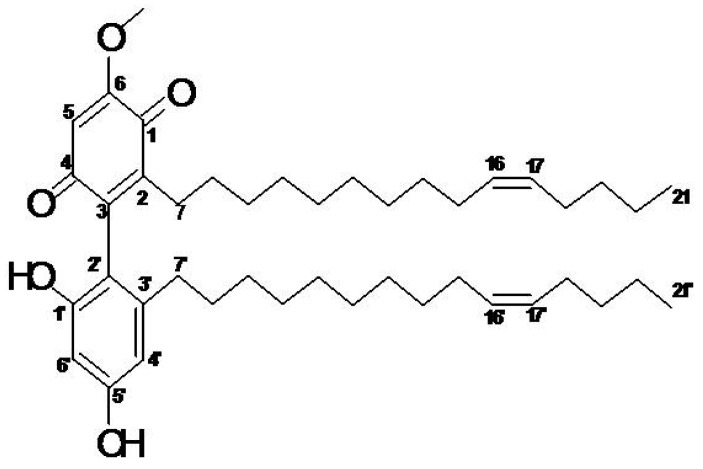
Chemical structure of demethylbelamcandaquinone B.

The identification of the compound was performed based on its MS and NMR spectral data. As determined from its molecular ion [M + H]^+^ peak at *m/z* 663.4883 (Figure 1), the compound of interest was identified as demethylbelamcandaquinone B (Dmcq B, Figure 2) with a molecular formula of C_43_H_66_O_5_.

### 2.2. Promotion of Viable Cells with M. pumilum var. alata Treatments

**Figure 3:**
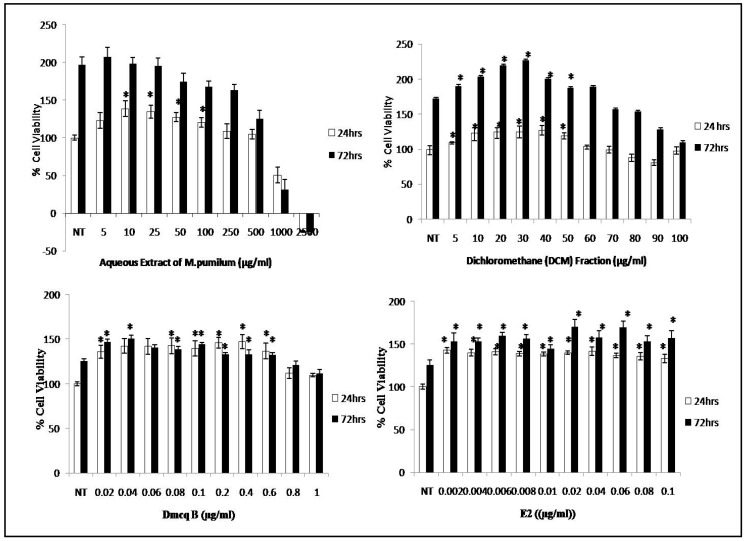
Stimulation effects of *M. pumilum* var. *alata* crude aqueous extract, DCM fraction, Dmcq B and E2 (positive control) on osteoblast by MTS cell proliferation assay. Percentage of viable cells were increased at concentration ranging from 10–100 µg/mL for 24 h but declined at concentrations of more than 1000 µg/mL for crude *M. pumilum* var. *alata* extract-treated cells whereas cell viability was increased at concentration of 10–50 µg/mL for DCM fraction after 24 and 72 h treatment. Interestingly, Dmcq B isolated from DCM fraction exhibited a higher percentage of viable cells at low concentrations (0.02–0.6 µg/mL) where as for the positive control (treated with E2), the percentage of viable cells was significantly increased at concentrations of 0.002 to 0.08 µg/mL after 24 and 72 h treatments. The data are presented as means of percentage of treatment ± SEM; *n* = 6. * Significantly increased (*p* < 0.05) compared to non-treated group (NT).

After 24 h treatment with crude aqueous extract of *M. pumilum* var. *alata* at the concentrations of 10–100 µg/mL, there was a significant increase in the viability of pre-osteoblasts (Figure 3). The crude extract was further fractionated using three different solvents according to polarity. 

Among these fractions, the dichloromethane (DCM) fraction of crude aqueous extract demonstrated the highest percentage of viable cells at concentrations of 10 to 50 µg/mL after 24 and 72 h treatments, when compared to other fractions (Figure 3). A suspected active compound was identified from the DCM fraction based on the most prominent compound in the TLC results. 

Subsequently, the suspected active compound was isolated and further assessed by a cell viability assay. Interestingly, the compound of interest exhibited an increase of viable cells number at low concentrations (0.02–0.6 µg/mL) compared to crude extract and DCM fraction of *M. pumilum* var. *alata*. Meanwhile, the percentage of viable cells was significantly increased in the positive control (treated with 17-β estradiol or E2) at concentrations of 0.002 to 0.08 µg/mL (Figure 3). There were no significant differences among treatment groups between the time point of 24 h and 72 h after treatment. For the following experiments, the concentrations for each treatment were selected based on the optimization with Alizarin Red staining.

### 2.3. Modulation of Crude Extract, DCM Fraction and Dmcq B on Early Markers of Osteoblast Differentiation

**Figure 4:**
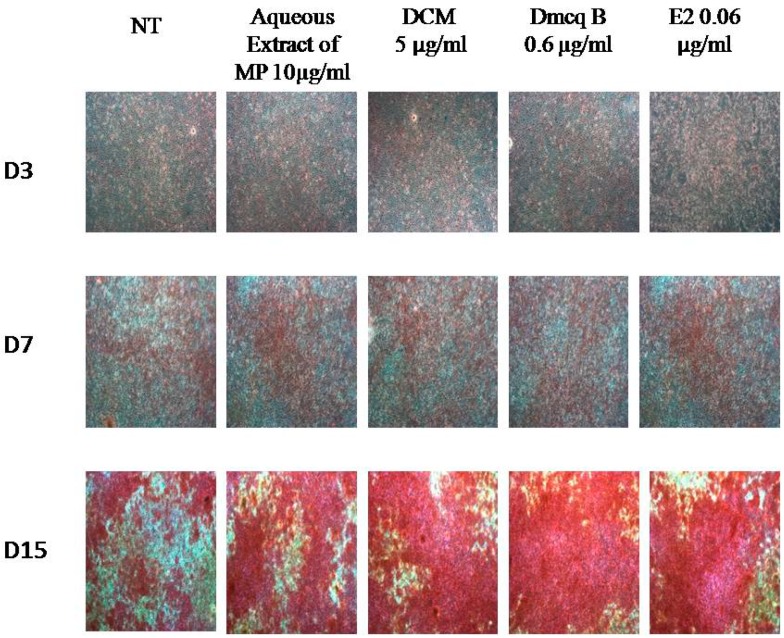
Effects of *M. pumilum* var. *alata* crude aqueous extract, DCM fraction, Dmcq B and E2 (positive control) on collagen synthesis during 15 days of differentiation induction. The photomicrographs were captured for all groups using an inverted microscope (magnification 100×). Collagen was stained intense pink. The stained-collagens were increased during differentiation. The collagen clusters were evenly distributed throughout the stimulated region in all *M. pumilum* var. *alata* treatments, resembling the expression in E2 treatment. NT non-treated group MP *M. pumilum* var. *alata* crude aqueous extract DCM dichloromethane fraction of crude aqueous extract Dmcq B demethylbelamcandaquinone B.

**Figure 5:**
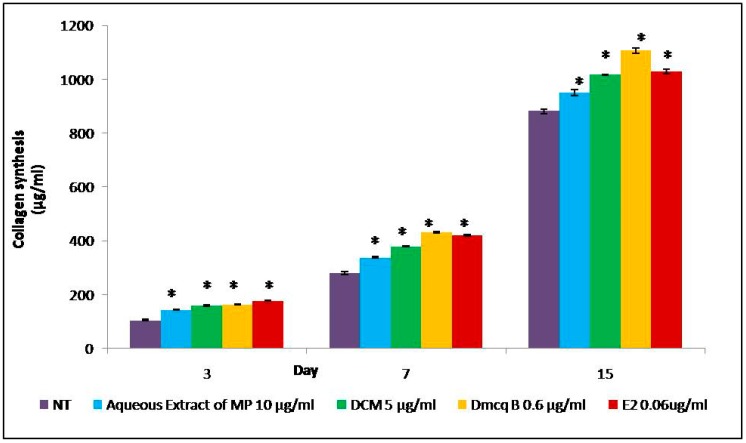
Collagen content from Sirius Red staining was measured at 15 days of differentiation induction. All *M. pumilum* var. *alata* treatments have significantly increased collagen content compared to non-treated group with ascending pattern, resembling the effects of E2 treatment. The data are presented as means of percentage of treatment ± SEM; *n* = 6. * Significantly increased (*p* < 0.05) compared to NT group at corresponding day of differentiation. NT non-treated group MP *M. pumilum* var. *alata* crude aqueous extract DCM dichloromethane fraction of crude aqueous extract Dmcq B demethylbelamcandaquinone B.

Collagen synthesis and ALP activity are considered as early-stage differentiation markers of the osteoblast phenotype. Sirius Red staining revealed the presence of collagen secreted by the osteoblasts, as indicated by the formation of intense pink cluster from the reaction between sulfonic acid in Sirius Red and collagen fibers. Microscopically, the pink clusters of collagen were more intense and evenly distributed throughout the stimulated region for all treatments at Day 15 compared to non-treated group (Figure 4). In line with this, all treatment groups developed an increase in collagen synthesis quantitatively in a time-dependent manner (Figure 5). 

**Figure 6:**
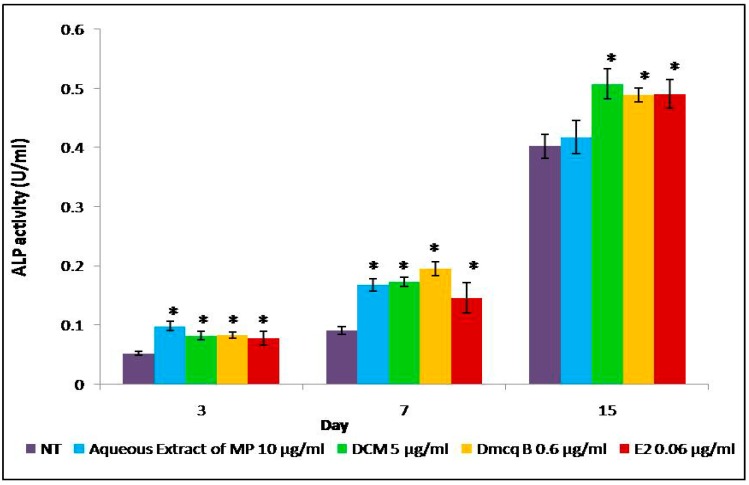
Effects of *M. pumilum* var. *alata* crude aqueous extract, DCM fraction, Dmcq B and E2 (positive control) on ALP activity during 15 Days of differentiation induction. DCM fraction and Dmcq B were significantly increased compared to non-treated group with an ascending pattern, resembling the expression with E2 treatment. Only *M. pumilum* var. *alata* crude extract was statistically increased at time points of Day 3 and 7. Then, the crude extract of *M. pumilum* var. *alata* did not attain a significant increase at Day 15. The data are presented as means of percentage of treatment ± SEM; *n* = 6. * Significantly higher (*p* < 0.05) compared to NT group at the corresponding day of differentiation. NT non-treated group MP *M. pumilum* var. *alata* crude aqueous extract DCM dichloromethane fraction of crude aqueous extract Dmcq B demethylbelamcandaquinone B.

This confirmed that *M. pumilum* var. *alata* treatments enhanced collagen synthesis which is the hallmark feature of early differentiated osteoblasts. The ALP activity for all treatments also showed similar trend to collagen synthesis analysis (Figure 6). These results further implied that *M. pumilum* var. *alata* treatments could facilitate osteoblast differentiation.

### 2.4. Modulation of Crude Extract, DCM Fraction and Dmcq B on Osteoblast Differentiation

**Figure 7:**
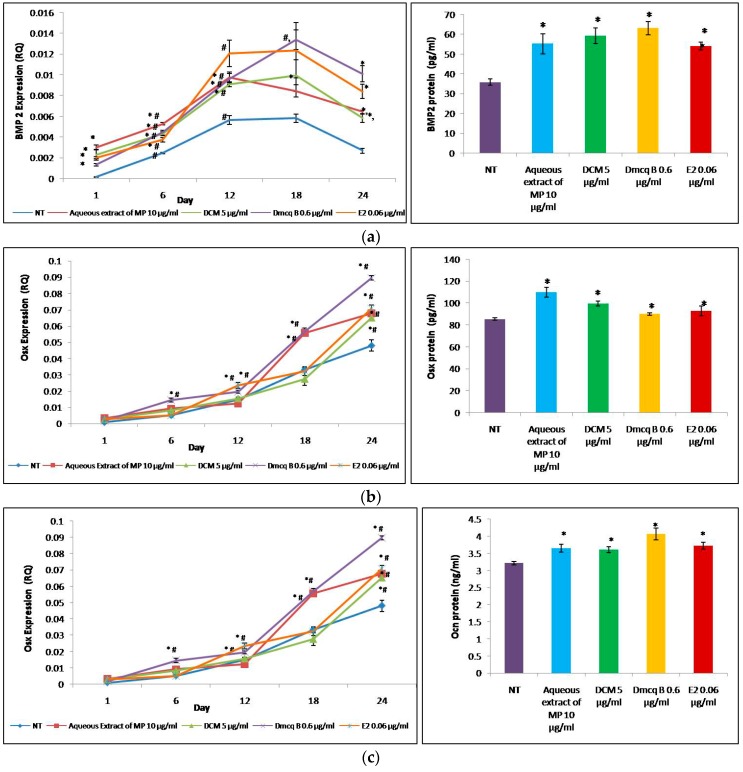
Effects of *M. pumilum* var. *alata* crude aqueous extract, DCM fraction, Dmcq B and E2 (positive control) on mRNA expressions of osteoblastic genes during 24 days of differentiation induction and protein expression at the corresponding day of maximal expression of mRNA. (**a**) BMP2 mRNA gradually increased time-dependently up to Day 12, maintained up to Day 18, and decreased to Day 24; (**b**) Osx mRNA gradually increased time dependently and peaked at Day 24; (**c**) Ocn mRNA is produced after Day 6 and peaked to the Day 18 and maintained to the Day 24 in all treatments compared to non-treated group, was peaked up only by the Day 24. Overall, the *M. pumilum* var. *alata* treatments showed significant increase in osteoblastogenesis associated genes, which at par with E2 treatment. E2-driven stimulation on gene expression at transcriptomic level was confirmed at protein level. The data are presented as means of percentage of treatment ± SEM; *n* = 6. * Significantly increased (*p* < 0.05) compared to NT group at a corresponding day# significantly increased (*p* < 0.05) compared to previous time-course. NT non-treated group MP *M. pumilum* var. *alata* crude aqueous extract DCM dichloromethane fraction of crude aqueous extract Dmcq B demethylbelamcandaquinone B.

The study showed that the studied genes sequentially expressed during osteoblast differentiation, which played critical roles in extracellular matrix formation and mineral deposition (Figure 7). 

In the present findings, BMP2 mRNA pattern was shown to be gradually increased in a time-dependent manner up to Day 12, maintained till Day 18, and decreasing to Day 24. This result showed that BMP2 is a signal for induction of osteoblast lineage commitment. Osx mRNA expression gradually increased in a time-dependent manner and peaked at Day 24. Osx is essential for osteoblast proliferation, differentiation and bone formation. Following the activation of BMP-2 and Osx mRNA from Day 1, the expression of OCN mRNA was produced after Day 6. The peak expression of OCN mRNA was observed at Day 18 for all treatment groups compared to non-treated group, which peaked only by Day 24. Osteocalcin, which only appears during the terminal phase of osteoblastic differentiation, binds to Ca^2+^ for bone mineralization. Overall, these treatments significantly elevated osteoblastogenesis-related genes during the 24 days period of culture. The effects of all treatments on transcriptomic activity of osteoblastogenesis-related genes were similar to that on protein expression.

### 2.5. Stimulation Effects of Crude Extract, DCM Fraction and Dmcq B in Mineralization

**Figure 8:**
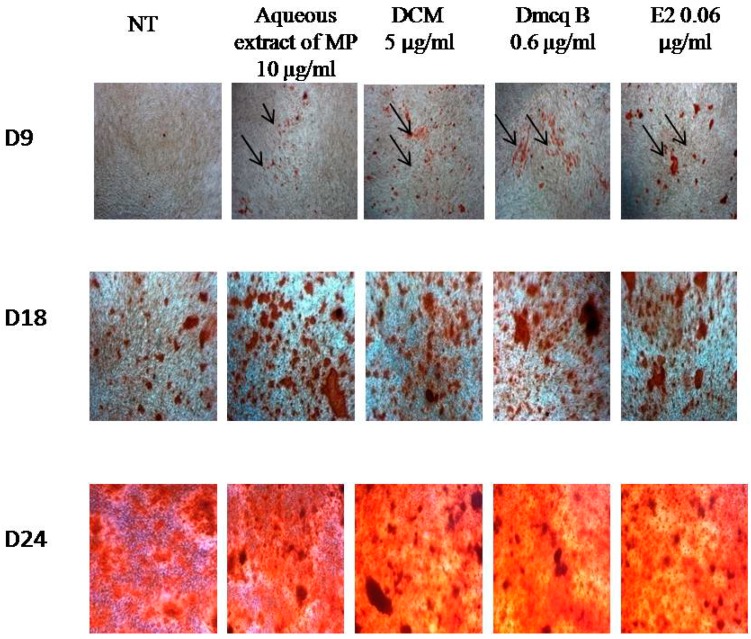
Effects of *M. pumilum* var. *alata* crude aqueous extract, DCM fraction, Dmcq B and E2 (positive control) on calcium deposition during the 24-days induction of differentiation. The photomicrographs were taken from all groups using an inverted microscope (magnification 100×). Calcium deposition was stained dark red. Following the expression of osteocalcin mRNA produced after Day 6, the stained-calcium deposited was increased during differentiation starting from the Day 9. The calcium deposition was evenly distributed throughout the stimulated region in all *M. pumilum* var. *alata* treatments, resembling the effects of E2 treatment. NT non-treated group MP *M. pumilum* var. *alata* crude aqueous extract DCM dichloromethane fraction of crude aqueous extract Dmcq B demethylbelamcandaquinone B.

Alizarin Red S staining exposed the presence of calcium in the bone nodules by forming a bright red colored alizarin red s-calcium complex, which acted as evidence of calcified regions for matrix mineralization (Figure 8). 

**Figure 9:**
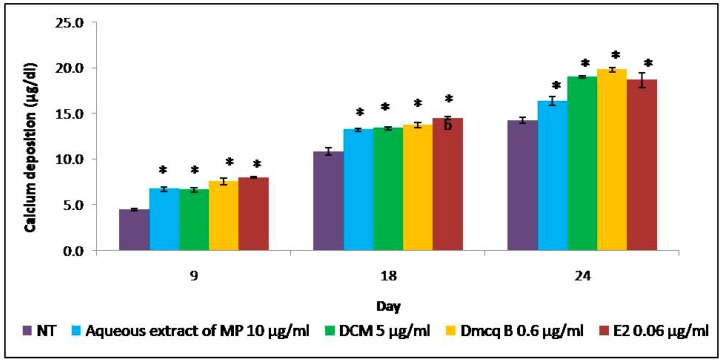
Calcium deposition was quantified during 24 days of differentiation induction. All the treatments of *M. pumilum* var. *alata* were significantly higher compared to non-treated group with an ascending pattern, resembling the expression with E2 treatment. The data are presented as means of percentage of treatment ± SEM; *n* = 6. * Significantly increased (*p* < 0.05) compared to NT group at corresponding day of differentiation. NT non-treated group MP *M. pumilum* var. *alata* crude aqueous extract DCM dichloromethane fraction of crude aqueous extract Dmcq B demethylbelamcandaquinone B.

The resulting Ocn mRNA pattern in this study showed that the gene was expressed after Day 6. Thus, the calcified regions were observed starting from Day 9. In parallel, the calcified regions were initiated on Day 9 in the all treatment groups compared to the non-treated group. All the treatments promoted more calcium deposits until Day 24 and hence increased mineralization as compared to non-treated group. This increased in calcium deposits was quantitatively confirmed colorimetrically with a calcium assay. The results clearly indicated enhanced deposition of calcium and accelerated mineralization of the matrix (Figure 9). In contrast to non-treated group, there were calcified nodules formations in a time-dependent manner in response to *M. pumilum* var. *alata* and E2 treatments.

### 2.6. Reduction of RANKL/OPG Ratio by Crude Extract, DCM Fraction and Dmcq B

**Figure 10:**
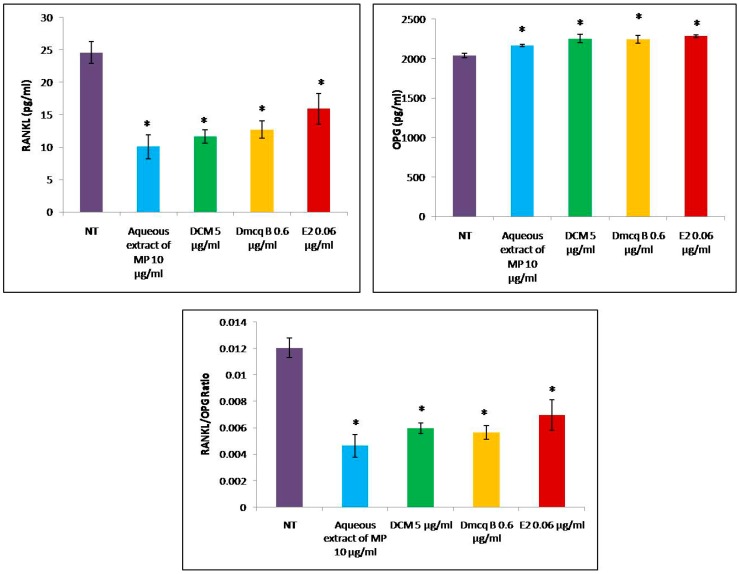
Effects of *M. pumilum* var. *alata* crude aqueous extract, DCM fraction, Dmcq B and E2 (positive control) on RANKL/OPG ratio. All *M. pumilum* var. *alata* treatments showed significantly lower RANKL protein expression followed by an increment of OPG protein expression, hence the RANKL/OPG ratio was decreased for all the treatments, resembling the effects of E2 treatment. The data are presented as means of percentage of treatment ± SEM; *n* = 6. * Significantly different (*p* < 0.05) compared to NT group. NT non-treated group MP *M. pumilum* var. *alata* crude aqueous extract DCM dichloromethane fraction of crude aqueous extract Dmcq B demethylbelamcandaquinone B.

As shown in Figure 10, all the treatments led to a significant decrease in the expression of RANKL protein in osteoblast cells. All treatments increased the secretion of OPG protein when compared to non-treated group (*p* < 0.05) and there were no significant differences among the treated groups. Both the RANKL/OPG ratio and RANKL protein levels were reduced with all treatments (Figure 10). Therefore, all the treatments might have exerted bone protective effects on osteoblast cells.

### 2.7. Effects of Crude Extract, DCM Fraction and Dmcq B on ER Protein Expression

**Figure 11:**
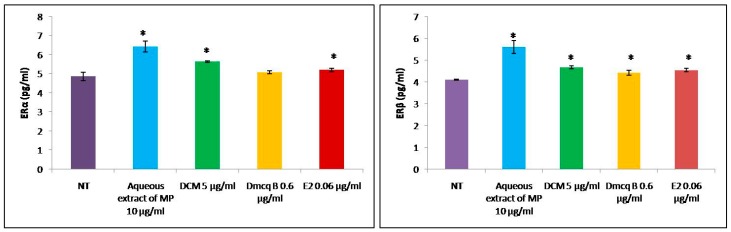
Effects of *M. pumilum* var. *alata* crude aqueous extract, DCM fraction, Dmcq B and E2 (positive control) on ERα and ERβ which were present on osteoblasts after 72 h of treatment. Our studies showed that *M. pumilum* var. *alata* crude extract and DCM fraction resembled E2 treatment, which significantly induced both ERα and ERβ protein expression. The active compound, Dmcq B, only led to an increase of ERβ protein expression and there was no significant difference in ERα protein expression when compared to normal control. * Significantly different (*p* < 0.05) compared to NT group. NT non-treated group MP *M. pumilum* var. *alata* crude aqueous extract DCM dichloromethane fraction of crude aqueous extract Dmcq B demethylbelamcandaquinone B.

As a positive control, E2 increased ERα and ERβ protein expressions in osteoblasts (Figure 11). This study showed that the *M. pumilum* var. *alata* crude aqueous extract and DCM fraction of crude aqueous extract were at par with E2 results in inductions of both ERα and ERβ protein expressions. Its active compound, Dmcq B only led to an increase of ERβ protein expression and there was no effect on ERα protein expression when compared to non-treated group. As a novel phytoestrogen isolated from *M. pumilum* var. *alata*, it can be reasonably speculated that Dmcq B would stimulate osteogenic effects through ERβ signaling.

## 3. Discussion

Phytoestrogens are plant-derived estrogenic compounds with similar molecular structures to estrogen that can be isolated from a variety of plants and seeds. Recent studies have demonstrated phytoestrogens’ osteogenic effects and good safety profile, making them a potential therapeutic option for menopausal osteoporosis. Most of the phytoestrogens identified so far have phenolic structures, which might serve as agonists of ERs [34]. Demethylbelamcandaquinone B (Dmcq B) was found to be the most abundant compound in the DCM fraction of *M. pumilum* var. *alata* crude aqueous extract, raising the possibility that Dmcq B was the critical phytoestrogen compound in the DCM fraction, which has potential anabolic bone effects. In addition, other quinone compounds isolated from different plants proved to be beneficial for the treatment of osteoporosis. For instance, anthraquinone isolated from *Morinda officinalis* was found to inhibit the process of bone resorption in vitro [35] and emodin from the plant genus *Rhamnus* [36] and thymoquinone of *Nigella sativa* [37] were shown to increase the proliferation and differentiation of osteoblast cells through BMP2 signaling pathway. On the other spectrum, Dmcq B was shown to have cardioprotective effects towards isoproterenol-induced myocardial infarction in rats [38].

The process of bone formation begins with initial increase in osteoblast viability followed by increased collagen content and enzymatic ALP activity, the development and maturation of the extracellular matrix, and mineralization associated with bone formation signaling pathway [39]. *M. pumilum* var. *alata* was recently reputed to exert potential osteogenic effects in in vivo studies. Nevertheless, the effects of crude extract of *M. pumilum* var. *alata* and its phytoestrogen compound(s) on osteoblast viability, early and mid-stage of differentiation, and mineralization have not been studied. The present study utilized MC3T3-E1 pre-osteoblast cells to analyze the osteogenic effect of these treatments at certain culture period. The present findings demonstrated for the first time, the direct stimulating effect of these treatments on MC3T3-E1 preosteoblasts, as evidenced by their ability in modulating osteoblast differentiation at the molecular level.

The present study results showed that DCM fraction of *M. pumilum* var. *alata* crude aqueous extract and Dmcq B at appropriate concentrations could promote the proliferation of osteoblasts, predominantly, on the third day of osteoblast cultures. Moreover, in this study, the crude extract, DCM fraction and Dmcq B showed no cytotoxic effects on osteoblast viability at any concentrations and, therefore, toxic side effects can be excluded. Collagen synthesis and ALP activity, the early phenotypic markers for mature osteoblasts, were all increased by *M. pumilum* var. *alata* treatments. Within the time frame of osteoblast proliferation and differentiation, collagen synthesis and ALP were expressed over the active matrix maturation phase (normally 3–15 Days) immediately following the cell proliferation period (about 1-3 Days). Therefore, in general, collagen synthesis and ALP activity were increased by *M. pumilum* var. *alata* treatments with the initiation of osteoblast differentiation [40].

All *M. pumilum* var. *alata* treatments on MC3T3-E1 cells resulted in increased expression of BMP2, which played a pivotal role in providing signal for commitment ofosteoblast lineage. This includes activation of collagen type I, ALP and osteocalcin expressions for osteoblast differentiation through transcription factors such as Runx-2 and Osx [41]. Our study showed that the expression of BMP2 mRNA peaked at Day 18, which was in line with the maximal expression of osteocalcin mRNA. BMP2 played an important role during osteoblast differentiation to regulate osteocalcin expression for mineralization process. Following that, our study showed a significant increment of Osx expression, an indispensable transcription factor during osteoblast differentiation with the maximal stimulatory effect at Day 24. This was in line with the findings of Zhuo et al. [42], which proved that other than osteoblast differentiation, Osx was also involved in the formation, maturation and function of osteocytes, when some differentiated osteoblasts turn into osteocytes [43]. Osteocalcin (Ocn), a phenotypic marker for the later stage of osteoblast differentiation [44], was produced after Day 6 and was shown to be increased by all treatments, consistently up to the Day 18. Apart from the significant increase in osteocalcin expression, the high mineralization process further demonstrated the anabolic effects of all *M. pumilum* var. *alata* treatments. BMP2 has been shown to stimulate bone formation by activating Osx which regulates expression of osteogenic genes such as collagen type Ι, ALP and Ocn [41]. During osteoblast differentiation, osteoblasts synthesize and secrete an organic matrix, the osteoid, predominantly formed by collagen type I [45]. During mineralization, the high activity of alkaline phosphatase induces the release of inorganic phosphorus and increase the local concentration of inorganic phosphate to promote mineralization [46]. Later on, differentiated osteoblasts produce Ocn, a protein that binds calcium, thereby contributing to the increase in calcium deposition. The formation of crystal hydroxyapatite results in the mineralization of the organic matrix [47]. The crude aqueous extract of *M. pumilum* var. *alata*, DCM and Dmcq B were at par with E2 treatment in inducing osteogenic differentiation from the early to late phases.

Futhermore, differentiated osteoblasts also expressed RANKL and OPG, the markers of osteoblast-regulated osteoclast activation and bone resorption process [48]. RANKL is an essential regulator for activation, differentiation and survival of osteoclasts by binding to the specific RANK receptor, which is found on the surface of osteoclast precursors and mature osteoclasts [49]. On the other hand, OPG is a decoy receptor of RANKL and thus acts as an inhibitor of bone resorption. All *M. pumilum* var. *alata* treatments were shown to exhibit reduction of RANKL expression and RANKL/OPG ratio. Thus, *M. pumilum* var. *alata* crude aqueous extract, DCM fraction of crude aqueous extract and Dmcq B as well as E2 treatment protected bone not only by improving osteoblast differentiation, but also by suppressing the osteoclast-mediated bone resorption.

Crude aqueous extract, DCM fraction of crude aqueous extract and Dmcq B were shown to slightly exhibit the expression of ER proteins. In physiological milieu, both ERα and ERβ are found on osteoblastic cells. ERα is mainly expressed in cortical bone and essential for longitudinal growth, whereas ERβ is predominantly present in cancellous bone and is important for the maintenance of bone substance [50,51]. Matched cell cultures showed that both E2 and Dmcq B promoted osteoblast proliferation as well as differentiation, possibly through ER signaling pathway. Dmcq B, a compound with a chemical core reminiscent to conventional estrogenic ligands [52,53], is similar to that of genistein and quercetin, which are universally known as phytoestrogen [54]. Thus, Dmcq B has the potential to exert endogenous estrogen action which may generate osteoanabolic effects. Further studies are necessary to specify the role of ER in Dmcq B-induced osteoblastic differentiation via interactions between classical and non-classical ERs in genomic and non-genomic responses.

Based on the present study, there are two possible mechanisms underlying the bone protective effects of Dmcq B during osteoblast differentiation. Firstly, Dmcq B may directly activate BMP signaling by interacting with Smads phosphorylation. Secondly, Dmcq B may indirectly activate BMP2 expression through ER signaling pathway. This is because endogenous estrogen was proven to be capable of directly stimulating the BMP2 pathway [55].

## 4. Materials and Methods

### 4.1. Isolation and Identification of M. pumilum var. alata Active Compound

The following instruments were used: ATR-FTIR GX (PerkinElmer, Waltham, MA, USA), HRESI-MS MicrOTOF-Q 86 (Bruker, Billerica, MA, USA) and FT-NMR 600 MHz Cryoprobe Bruker Avance III (Billerica, MA, USA). The following adsorbents were used for fractionation and purification, including Merck Si-gel 60 (40–63 µm, Cat. No. 1.09385) for column chromatography (CC), Sephadex LH-20 for gel permeation chromatography and Merck Kieselgel 60 F254.025 mm (Cat. No. 1.05554) for thin layer chromatographic (TLC) analysis. Solvents used were analytical grade and purchased from Merck (Darmstadt, Germany). 

The voucher specimen of *M. pumilum* var. *alata* is UKMB 30006/SM 2622 which was deposited in the Herbarium of Universiti Kebangsaan Malaysia. The air dried and powdered leaves of *M. pumilum* var. *alata* (8720 g) were extracted at the temperature of 60 °C for 2 h with a ratio of dried leaves-water of 1:30, followed by freeze drying to obtain powdered *M. pumilum* var. *alata* crude aqueous extract with the a net yield of 700 g. Part of the crude aqueous extract (200 g) was then successively fractionated with hexane, dichloromethane (DCM) and methanol (MeOH) solvents using the reflux method according to their boiling point temperature for 4 h. Based on cell viability assay, the fractions were separated using thin layer chromatography (TLC); from which the compounds profile was compared and the active compound was identified from the DCM fraction (data not shown). The DCM fraction (1.3 g) was then subjected to silica gel column chromatography and eluted with a gradient system of chloroform (ChCl_3_)-ethyl acetate (EtOAc) to yield 15 fractions. Sub-fraction F8 (81.9 mg) was subjected to Sephadex LH-20 column chromatography and eluted with 1% methanol in chloroform as mobile phase to yield ten sub-fractions. Sub-fractions F9F and F10F (53.6 mg) were further purified using silica gel with a ratio of CHCl_3_–EtOAc (9:1, *v*/*v*) to yield a bioactive compound which was identified as demethylbelamcandaquinone B (Dmcq B) based on its UV, FTIR, MS and NMR data and comparison with literature values. Purity of the compound was more than 98%, based on their physicochemical properties, NMR and HRESI-MS data. Demethylbelamcandaquinone B: 35.0 mg; dark orange and waxy (CHCl_3_); UV (EtOH) λ_max_ nm (log ε): 213 (3.16), 275 (3.04); IR (ATR) ν_max_, cm^−1^: 3275, 2922, 2854, 1680, 1638, 1618, 1600, 1456, 1339, 1226, 1147, 1051, 847, 722; positive HRESI-MS *m/z*: 663.4883 [M + H]^+^ (C_43_H_66_O_5_, MW 662.9811); ^1^H-NMR (600 MHz; CDCl_3_) δ_H_ (ppm): 0.89 (6H, *m*, H-21, H-21′), 1.33–1.18 (18H, *m*, H-8-H-14, H-19-H-20, H-8′-H-14′, H-19′-H-20′, *overlapped*), 2.01 (8H, *m*, H-15, H-18, H-15′, H-18′), 2.18 (H, *m*, H-7) 2.35 (*m*, H-7), 2.23 (2H, *m*, H-7′), 3.85 (*s*, OMe), 5.34 (4H, *m*, H-16-H-17, H-16′-H-17′), 5.99 (*br s*, OH), 6.16 (*d*, *J* = 1.8 Hz, H-6′), 6.29 (*d*, *J* = 1.8 Hz, H-4′); ^13^C-NMR (150 MHz; CDCl_3_) δ_C_ (ppm): 14.0 (C-21, C-21′), 22.4 (C-20, C-20′), 26.9 (C-18, C-18′), 27.2 (C-15, C-15′), 28.2 (C-7), 33.5 (C-7′), 29.0-30.0 (C-8–C-14), (31.8 (C-19), 32.0 (C-19′), 56.3 (OMe), 129.9 (C-16-C-17, C-16′-C-17′), quinone ring; 182.2 (C-1), 146.9 (C-2), 141.0 (C-3), 188.0 (C-4), 107.4 (C-5), 158.9 (C-6), benzene ring; 153.7 (C-1′), 112.2 (C-2′), 143.2 (C-3′), 108.2 (C-4′), 156.7 (C-5′), 100.9 (C-6′). The active compound was further assessed with viability cell assay.

### 4.2. Cell Culture

Murine pre-osteoblastic MC3T3-E1 cells (ATCC, Rockville, MD, USA) were grown in α-modified minimal essential medium with 10% heat-inactivated fetal bovine serum and 1% antibiotic-antimycotic (Gibco Life Technologies, Inc., Grand Island, NY, USA) at 37 °C in 5% CO_2_ atmosphere. After the cells reached confluence, they were cultured in differentiation medium α-MEM containing 50 μg/mL ascorbic acid (AA) and 10 mM β-glycerophosphate (GP) (Sigma, St. Louis, MO, USA) to induce osteoblast differentiation for 2 to 24 days at various doses of treatments. For estrogen receptor protein expression, 10% charcoal-dextran-treated fetal bovine serum (CD-FBS) (Hyclone Laboratories Inc., Logan, UT, USA) was used to replace heat-inactivated fetal bovine serum.

### 4.3. Determination of Cell Viability

Cell viability was tested using the MTS (3-(4,5-dimethylthiazol-2-yl)-5-(3-carboxy-methoxyphenyl)-2-(4-sulphenyl)-2*H*-tetrazolium) assay (Promega, Madison, WI, USA). Crude aqueous extract of *M. pumilum* var. *alata*, hexane, DCM and MeOH fractions of aqueous extract, demethylbelamcandaquinone B (Dmcq B) and 17-β estradiol (E2-positive control) were analysed for their proliferative effects on pre-osteoblast cells. Fractions and active compounds were dissolved in 1.0 mL dimethylsufoxide. The final culture concentration of dimethylsufoxide (Sigma, St. Louis, MO, USA) was kept around 0.1% to 0.001% to reduce its influence on the cells. 17-β estradiol was dissolved in ethanol with the final concentration of ethanol ranging from 0.05% to 0.001%. During the experiment, stock solutions were diluted at various concentrations with the differentiation media. Cells were plated at a density of sterile 1 × 10^4^ in 96-well plate and incubated overnight. After the first 24 h, the medium was discarded and applied with differentiation media containing various concentrations of *M. pumilum* var. *alata* crude extract, its fraction and active compound extract. After incubation, a total of 120 µL of differentiation medium and MTS mixture (100 µL differentiation medium and 20 µL MTS) was added to each well and incubated for 2 h. The absorbance of MTS formazan formed was determined spectrophotometrically at 490 nm with a microtiter plate reader (Tecan, Grödig, Austria). The viability assay was performed to obtain the optimum dose of these treatments for subsequent experiment. The proliferation of pre-osteoblasts was measured at 24 and 72 h. The quantity of formazan products is proportional to the number of viable cells in the culture.

### 4.4. Collagen Content

The amount of collagen was observed and quantified using a Sirius Red dye assay, with slight modification of the method described by Kim et al. [56]. A total of 5 × 10^3^ cells/well were seeded in a 24-well plate. Treatments with differentiation medium were carried out once every three days. Staining and quantity of collagens were measured on Day 3, 7 and 15. After treatments, cell was washed with PBS, followed by addition of 500 µL Sirius Red (Sigma, St. Louis, MO, USA)) solution, which was diluted to 0.1% in saturated picric acid(Sigma, St. Louis, MO, USA)) to the culture plates and incubated for 1 h in dark. The cells were rinsed with 1 mL of 0.01 M HCL (three times) until the solution was colourless. The dark red collagen cluster formed was observed under an inverted microscope (Olympus, Tokyo, Japan). Following that, the samples were added with 500 µL of 0.1 M NaOH to elute the red colour dye. Then, 100 µL red-dye eluents were transferred to 96-well plates and the absorbance was read at 540 nm using a microplate reader. The absorbance was calculated to collagen content (unit in µg/mL) according to a standard curve. For standard collagen, collagen stock solution (Gibco Life Technologies, Inc.) at the concentration of 3450 µg/mL was diluted with PBS to a series of concentration with ratio 1:1. A total of 100 µL for each concentration were transferred to 96-well microplate, and incubated 37 °C for several days until it was completely dried. The dried remnants were subjected to Sirius Red assay as described above, to obtain a standard curve.

### 4.5. ALP Activity

For alkaline phosphatase (ALP) activity measurement, 5 × 10^3^ cells/ well were seeded in 24-well plates. ALP activity was measured on Day 3, 7 and 15 using alkaline phosphatase assay kit (Abcam Inc., Cambridge, UK). Treatments with differentiation medium were carried out once every three days. The total ALP activity was measured using p-nitrophenylphosphate (pNPP) as substrate and quantified colorimetrically at 405 nm.

### 4.6. Quantitative Analysis of Gene Expression by Real-Time RT-PCR

To understand the underlying molecular pathway for the osteogenic effects of *M. pumilum* var. *alata* and its active compound, the quantitative analysis of gene expression was conducted at time points of Day 1, 3, 6, 12, 18 and 24. Briefly, 2 × 10^4^ cells/well were seeded in 24-well plates. After treatments, the cell pellet was lysed in TRI-Reagent solution (Molecular Research Center, Cincinnati, OH, USA) and stored at 80 °C until further analysis. Total RNA extraction was done in an RNase-free environment, according to the manufacturer’s instructions. Genes and forward/reverse primers used for RT-PCR were designed using the National Centre for Biotechnology Information (NCBI) website for the following genes, BMP2, Osx and Ocn. Primer sequences are listed in Table 1. Accordingly, single-stranded cDNA was synthesized from 100 ng of total RNA using an iScript cDNA synthesis kit (BioRad Laboratories, Hercules, CA, USA). Each 20 µL aliquot contained 1 µL of RNA, 4 µL of 5x iScript reaction mix and 15 µL of ultrapure water. The reaction mix was incubated for 5 min at 25 °C, 30 min at 42 °C, and 5 min at 85 °C to obtain the cDNA template. Real-time PCR was performed in an iCycler iQ5 Real-Time PCR Detection System (Bio-Rad Laboratories, Hercules, CA, USA). The genes were amplified with a 20 µL of reaction mix consisting of iQ SYBR Green Supermix, cDNA template and primers. Initial denaturation of DNA was carried out at 95 °C for 3 min, 60 cycles amplification for the denaturation (95 °C, 10 s) and annealing and extension (55 °C, 30 s). The data collection and real-time analysis were performed at 95 °C for 1 min and 55 °C for 1 min, respectively. The PCR products were resolved on 2% agarose gel electrophoresis. In all cases, single bands of the expected size were observed. The specificity of each PCR product was further performed by melting curve analysis. The relative amount of gene expression was normalized with internal control of β-actin and calculated according to the following formula:

Relative Quantitation (RQ) Ct ^β-actin^ − Ct ^target gene^ (1)

where Ct = the cycle number at threshold level.

### 4.7. Enzyme-Linked Immunosorbent Assay (ELISA)

ELISA kits were used to detect BMP2, Osx, Ocn, ERα and ERβ according to the manufacturer’s instruction (Elabscience Biotechnology, Wuhan, China). Briefly, 2 × 10^4^ cells were treated with various concentrations of treatments for the indicated times (for osteoblastic genes: the day at which the highest amount of gene has been expressed, Osx-Day 24, BMP2 and Ocn-Day 18; for ER genes: after 72 h of treatment when cell proliferation was arrested). The culture cells pellet was then lysed using freeze-thaw method for three times. The standard and samples were transferred to a 96-well microtiter plate coated with the detective antibody and incubated for 90 min at room temperature. After washing three times with washing buffer, horseradish peroxidase conjugated streptavidin was added to each well successively and incubated. Horseradish peroxidase catalyzed the conversion of a chromogenic substrate to a colored solution. The absorbance of each well was photometrically determined using a microplate reader (Tecan, Grödig, Austria) at 405 nm. The content of protein was calculated according to standard curve.

### 4.8. Mineralization

Calcified nodule formation was assessed using Alizarin Red S staining. Briefly, 5 × 10^3^ cells/well were seeded in 24-well plate in differentiation medium. Treatments were given at Day 9, 18 and 24 and the medium was changed every three days. At the end of the treatments, cells were washed with phosphate-buffer saline (PBS) and then fixed with 10% formaldehyde for 15 min. Cells were then washed with PBS and exposed to Alizarin Red S solution (pH 4.1–4.3) (Sigma) for 30 min at room temperature. Bright red calcified nodules were then photographed and quantification was done using calcium assay kit (Chemicon International, Temecula, CA, USA). For quantification of insoluble calcium in the matrix layer of the cells, the cultures were decalcified with 0.6 M HCL. The soluble calcium in the supernatant was measured with at wavelength of 612 nm with a microtiter plate reader (Tecan, Grödig, Austria).

### 4.9. Statistical Analysis

All of the data were statistically analyzed using Statistical Package for Social Sciences (SPSS) software version 20 (IBM, New York, NY, USA). The normality of each test was performed using the Shapiro Wilk test. Normally distributed data were then tested with one-way ANOVA followed by Tukey’s HSD test as a post-hoc test. For data values that were not normally distributed, they were analyzed using Kruskal-Wallis and Mann–Whitney non-parameteric tests. All of the data presented as the mean ± standard error (SEM) from at least six replicates and *p* < 0.05 was considered statistically significant.

## 5. Conclusions

Collectively, we have demonstrated for the first time that a specific compound found in *M. pumilum* var. *alata* crude aqueous extract, namely demethylbelamcandaquinone B, exerted osteoanabolic effects via its potential action on ER in osteoblasts. The detailed mechanisms of how Dmcq B stimulated BMP2 and ER signaling pathways require further studies.

## Figures and Tables

**Table 1:** Primers sequences for quantitative gene expression analysis.

Gene	Accession No	Primer Sequence (5′-3′)	Product Size (bp)
β-actin	NM_007393.5	F: gaagagctatgagctgcctga R: gcactgtgttggcatagaggt	185
BMP2	NM_007561.4	F: gtgccctggctgctatgg R: tgccgcctccatcatgtt	546
Osx	NM_130458.3	F: gcaagaggttcactcgctct R: gtggtcgcttctggtaaagc	110
Ocn	NM_031368	F: gcgctctgtctctctgacct R: aagcagggtcaagctcacat	181
